# Another advantage of multi-locus variable-number tandem repeat analysis that can putatively subdivide enterohemorrhagic *Escherichia coli* O157 strains into clades by maximum *a posteriori* estimation

**DOI:** 10.1371/journal.pone.0283684

**Published:** 2023-03-30

**Authors:** Shinichiro Hirai, Eiji Yokoyama, Naoshi Ando, Junji Seto, Kyoko Hazama, Keigo Enomoto, Hidemasa Izumiya, Yukihiro Akeda, Makoto Ohnishi

**Affiliations:** 1 Center for Emergency Preparedness and Response, National Institute of Infectious Diseases, Tokyo, Japan; 2 Division of Bacteriology, Chiba Prefectural Institute of Public Health, Chiba, Japan; 3 Department of Microbiology, Yamagata Prefectural Institute of Public Health, Yamagata, Japan; 4 Department of Bacteriology I, National Institute of Infectious Diseases, Tokyo, Japan; Universidad Nacional de la Plata, ARGENTINA

## Abstract

Enterohemorrhagic *Escherichia coli* O157 (O157) strains can be subdivided into clades based on their single-nucleotide polymorphisms, but such analysis using conventional methods requires intense effort by laboratories. Although multi-locus variable-number tandem repeat analysis (MLVA), which can be performed with low laboratory burden, has been used as a molecular epidemiological tool, it has not been evaluated whether MLVA can be used the clade subdivision of O157 strains like it can for that of other pathogenic bacteria. This study aimed to establish a method for subdividing O157 strains into clades using MLVA data. The standardized index of association, *I*^S^_A_, for O157 strains isolated in Chiba prefecture, Japan (Chiba isolates) revealed the presence of unique tandem repeat patterns in each major clade (clades 2, 3, 7, 8, and 12). A likelihood database of tandem repeats for these clades was then constructed using the Chiba isolates, and a formula for maximum *a posteriori* (MAP) estimation was constructed. The ratio of the number of O157 strains putatively subdivided into a clade by MAP estimation from MLVA data relative to the number of O157 strains subdivided using single-nucleotide polymorphism analysis (designated as the concordance ratio [CR]) was calculated using the Chiba isolates and O157 strains isolated in Yamagata prefecture (Yamagata isolates). The CRs for the major Chiba and Yamagata isolate clades, other than clade 2, were 89%–100%. Although the CR for clade 2 Chiba isolates was >95%, that of the Yamagata isolates was only 78.9%. However, these clade 2 CRs were not significantly different from one another, indicating that clade 2 strains can be subdivided correctly by MAP estimation. In conclusion, this study expands the utility of MLVA, previously applied predominantly for molecular epidemiological analysis, into a low-laboratory-burden tool for subdividing O157 strains into phylogenetic groups.

## Introduction

In the ongoing surveillance of enterohemorrhagic *Escherichia coli* O157 (O157) infections by Japanese public institutes, multi-locus variable-number tandem repeat analysis (MLVA) has been widely used as a molecular epidemiological tool [1–3]. However, the additional potential use of MLVA as a method for subdividing O157 strains into clades, i.e., phylogenetic groups, has not been investigated [4–6]. The pathogenicity of O157 strains differs depending on their clade, with the most pathogenic strains belonging to clade 8 [7, 8]. Strains derived from a clade 8 clone emerged in various areas of Japan from 2007 to 2008 [9]. Clade subdivision for O157 strains does not have high discriminatory power, unlike MLVA [10, 11], but it can evaluate the pathogenicity of O157 clones emerging in areas. Therefore, surveillance of the distribution of O157 clades in an area is useful to estimate the public health risk posed by O157 infections [11].

At present, surveillance of O157 clade distribution has not been performed by most of the Japanese public institutes. The main reasons for this are likely the high burdens of laboratory work and cost of investigating clades by using single-nucleotide polymorphisms (SNPs) at 32 loci [5] or lineage-specific polymorphism assay-6 (LSPA-6) [12, 13]. Yokoyama et al. [14] adapted a low-cost amplification-refractory mutation system (ARMS)-PCR method to subdivide O157 strains into clades, but this method is not suitable for use in large-scale studies because it requires agarose gel electrophoresis of the PCR products. Etoh et al. [7] developed a convenient high-resolution melting analysis (HRM) method that runs on a real-time PCR apparatus to investigate SNPs in a large number of O157 strains. However, the cost of HRM (approximately 8.5 US$/strain) is almost three times higher than that of ARMS-PCR (approximately 3.0 US$/strain).

A possible solution for the problem of the excessive laboratory burden and cost associated with classifying O157 strains may be the use of MLVA [6]. Our previous study [4] analyzed O157 strains by IS-printing, which is a molecular epidemiological tool to investigate the distribution of insertion sequence *629* in the genome [15], and it found that most strains belonging to the same clade showed a unique IS-printing pattern, i.e., linkage disequilibrium of the insertion sequence in each clade. The result suggested that similar biased distribution of another molecular epidemiological tool, MLVA, might be observed. In fact, a previous study of another pathogen, *Mycobacterium tuberculosis*, reported that the organism showed a biased distribution of MLVA data in each clade and such distribution could be used to subdivide the organism into phylogenetic groups by maximum *a posteriori* (MAP) estimation based on Bayes’ theorem [6, 16, 17]. Because MLVA can be performed by multiplex PCR with a relatively low laboratory burden [2, 3], it is routinely performed in Japanese surveillance for O157 strains by public institutes [1]. Thus, if MLVA for O157 could also be used by the institutes for the subdivision of O157 strains into clades, it would remove the burdens of both laboratory work and cost for obtaining this important information.

Therefore, this study aimed to determine whether MLVA could be used to successfully subdivide O157 strains into clades. We investigated the linkage disequilibrium in the MLVA data for each clade using O157 strains isolated in a single area (Chiba prefecture) and constructed a MAP estimation formula to subdivide the O157 strains into clades. Using this new formula, the concordance ratio (CR) was calculated from the number of O157 strains subdivided into the correct clade by the MAP estimation compared with the number of strains subdivided appropriately by SNP analysis [18] and LSPA-6 [12, 13] for the strains isolated from remote two areas (Chiba and Yamagata prefectures).

## Materials and methods

### Bacterial strains

From the O157 strains isolated from human stools in 2018–2019 in Chiba prefecture (Chiba isolates) and in 2002–2007 in Yamagata prefecture (Yamagata isolates), a subset of epidemiologically unlinked strains was selected. In accordance with the Act on Prevention of Infectious Diseases and Medical Care for Patients Suffering Infectious Diseases (Act No. 114 of 1998), O157 infections in Japan are reported to local public health institutes, which then conduct standard epidemiological studies. The results of these studies are used to classify the O157 infections as sporadic cases, outbreak cases, or intra-family cases. For the present study, we included all the O157 strains that were isolated from sporadic cases; additionally, when multiple O157 strains were isolated in an outbreak or an intra-family scenario, the O157 strain that was isolated first was selected. In total, 136 Chiba isolates and 81 Yamagata isolates were selected as epidemiologically unlinked O157 strains. DNA was extracted from each strain using InstaGene matrix (Bio-Rad, Hercules, CA, USA), diluted to a concentration of 2 ng/μl, and used for SNP, LSPA-6, and MLVA analyses.

### Subdivision of O157 strains into clades

In this study, Chiba isolates were subdivided into clades for the first time by using the data from the SNP analysis [18] and LSPA-6 [12, 13] (S1 Table), as previously reported [4]. The SNP analysis determined six SNPs in the O157 genome by ARMS-PCR [14]. LSPA-6 subdivided O157 strains into three lineages by investigating alleles of six genes using PCR [19]. Yamagata isolates had also previously been subdivided in a previous study [18], and these were also used for this study (S1 Table).

### MLVA for O157 strains

The Chiba and Yamagata isolates were analyzed by MLVA for 17 loci, which differs slightly from the MLVA for 18 loci used in a previous study [3]. In the present study, one of the 18 loci, “O157-10”, was excluded from the MLVA because TR diversity of this locus was much higher than those of the other loci [3], potentially causing the TRs in O157-10 to change more frequently. For two of the 17 loci (“EH111” and “EH157-12”), primer sequences described in a recent study were used to prevent nonspecific amplification by multiplex PCR in the MLVA [20]. The MLVA used in this study has been developed to analyze three serotypes (i.e., O157, O26, and O111) of enterohemorrhagic *E*. *coli* [3]. Therefore, some of the 17 loci are serotype-specifically absent among all or most strains in each of the three serotypes. In this study, when there was no amplification of a locus by multiplex PCR in the MLVA, the TR in the locus was designated as “0” instead of as “−2”. To calculate MAP, even if there was no amplification by multiplex PCR in the MLVA, one allele needed to have a value of “0.”

### Analyses of minimum spanning tree and linkage disequilibrium of TR patterns obtained from MLVA

To investigate differences in the TR patterns observed in the O157 strains in each clade, a minimum spanning tree (MST) analysis was carried out using MLVA-Mate software (released March 2019) [21]. MLVA data for the Chiba isolates were imported into the software, and an MST was reconstructed using the default settings. MLVA-mate and its manual have been reposited on the website of National Council of Local Public Health Institutes in Japan (https://www.chieiken.gr.jp/slink.html#manuals) [22].

For the Chiba isolates, the occurrence of linkage disequilibrium in the TR patterns of the MLVA data for each clade was evaluated using the standardized index of association (*I*^S^_A_) [23]. LIAN Ver. 3.6 software was used to calculate the *I*^S^_A_ for each clade from the ratio of the variance of observed mismatches in the test set (*V*_*D*_) to the variance expected for a state of linkage equilibrium (*V*_*e*_), scaled by the number of loci used in the analysis (*l*), from:

$$I_{A}^{S}=\frac{1}{l-1}\left(\frac{V_{D}}{V_{e}}-1\right)$$

The significance of the ratio of *V*_*D*_ to *V*_*e*_ was determined by Monte Carlo simulations with 10^3^ resamplings.

### Construction of a MAP estimation formula for putative subdivision of O157 strains into clades using MLVA data

To putatively subdivide the O157 strains into clades based on the TR patterns in the MLVA data, we used a MAP estimation based on Bayes’ theorem. Several previous studies [24–26] developed the method for bacterial identification using MAP estimation, and one of these studies [26] presented the formula as follows:

$$P\left(t_{i}|R\right)=\frac{P\left(t_{i}\right)P(R|t_{i})}{\sum_{i}^{}P\left(t_{i}\right)P(R|t_{i})}$$

where *P*(*t*_*i*_|*R*) denotes the posterior probability that an organism giving test results *R* is a member of taxon *t*_*i*_, *P*(*t*_*i*_) represents the prior probability of the taxon, and *P*(*R*|*t*_*i*_) is the likelihood that a member of taxon *t*_*i*_ will yield test results *R*. On the basis of the formula presented by that previous study [26], we constructed new formulas to subdivide O157 in clades using MLVA data.

First, a likelihood database of TRs for each of the major clades (i.e., clades 2, 3, 7, 8, and 12) was constructed, using all Chiba isolates, from:

$$P\left(L_{n,N}|C_{i}\right)=\frac{R_{C_{i},N}}{R_{C_{2},N}+R_{C_{3},N}+R_{C_{7},N}{+R_{C_{8},N}+R}_{C_{12},N}}$$

where *n* is the locus number (i.e., EH111-11, EH111-14, …, O157-36, and O157-37 are 1, 2, …, 16, and 17, respectively); *N* is the number of TRs; *L*_*n*,*N*_ means that a strain has *N* number of TRs in locus number *n*; *i* is 2, 3, 7, 8, or 12; C_*i*_ is clade *i*; *P*(*L*_*n*,*N*_|*Ci*) is the likelihood of *L*_*n*,*N*_ for clade *i*; and *R*_*Ci*_,_*N*_ is the distribution rate of *N* among strains in clade *i*. The correspondence of parameters in formulas between our study and the previous study [26] was as follows: *L*_*n*,*N*_ corresponded to *R*; *C*_*i*_ to *t*_*i*_; *P*(*L*_*n*,*N*_|*C*_*i*_) to *P*(*R*|*t*_*i*_). Additionally, to correspond to the zero-frequency problem, distribution rates of all TRs were subjected to weighted smoothing [27]; i.e., the small value of “0.001” was added to each *R*_*Ci*_,_*N*_ in this study. To be more specific about the correspondence to the problem, the smoothing has an effect of preventing an inability to calculate MAP for atypical strains with TRs of likelihood “0” in the database. In addition, in this study, genome sequencing to confirm the absence of TRs in the loci was not performed for loci that could not be amplified by MLVA. However, the smoothing would have an additional effect of preventing subdivision into incorrect clades by MAP estimation, even if no amplification due to multiplex PCR failure occurred at a locus in the worst case.

Second, the posterior probability “*P*(*C*_*i*_|*L*_*n*,*N*_)” of clade *i* in *L*_*n*,*N*_ was calculated for each strain using the following formula:

$$P\left(C_{i}|L_{n,N}\right)=\frac{P(C_{i})P(L_{n,N}|Ci)}{P\left(C_{2}\right)P\left(L_{n,N}|C_{2}\right)+P\left(C_{3}\right)P\left(L_{n,N}|C_{3}\right)+\cdots +P(C_{12})P(L_{n,N}|C_{12})}$$

where *P*(*Ci*) is the prior probability, and *P*(*L*_*n*,*N*_|*C*_*i*_) is cited from the likelihood database. The *P*(*C*_*i*_|*L*_*n*,*N*_) in this study corresponds to *P*(*t*_*i*_|*R*) in the previous study [26].

Third, the *P*(*C*_*i*_|*L*_*n*,*N*_) in each of the 17 loci was calculated for each strain, and then all *P*(*C*_*i*_|*L*_*n*,*N*_) were continuously multiplied from:

$${PC}_{i}=\prod_{n=1}^{17}P\left(C_{i}|L_{n,N}\right)$$

The posterior probability “*P*(*C*_*i*_|*L*_*n*,*N*_)” of one locus in each clade was treated as the prior probability “*P*(*C*_*i*_)” of the next locus; e.g., *P*(*C*_*i*_|*L*_*2*,*N*_) is *P*(*C*_*i*_) of *n* = 3. *P*(*C*_*i*_) of the first MAP estimation was provided equal probabilities, i.e., 0.5 each, by the principle of insufficient reason. Finally, the clade of the strain was putatively identified as the value of *i*, for which *PC*_*i*_ is the maximum among *PC*_*2*_, *PC*_*3*,_ *PC*_*7*_, *PC*_*8*,_ and *PC*_*12*_.

### CR of clade subdivision using MAP estimation versus SNP and lineage analyses

CRs were calculated to evaluate whether the Chiba and Yamagata isolates were correctly subdivided into clades by MAP estimation, based on MLVA data, from:

$${CR}_{i}\left(\%\right)=\frac{{NP}_{x}}{{NC}_{x}}\times 100$$

where *CR*_*i*_ is the CR for O157 strains in clade *i*; *NC*_*x*_ is the number of O157 strains subdivided into clade *x* by SNP analysis and LSPA-6; *NP*_*x*_ is the number of clade *x* strains putatively subdivided into clade *x* by MAP estimation from MLVA.

### Statistical analysis

Differences among the CRs of the Chiba and Yamagata isolates were compared by performing a Chi-squared test in js-STAR 2019 release 9.8.7j software [28]. A value of *p* < 0.05 was considered to indicate a significant difference. If an O157 strain with homoplasy was included in the likelihood database, the strain was likely to be correctly subdivided into a clade by MAP estimation. Chiba isolates were used to construct the database, but Yamagata isolates were not. Therefore, a statistical analysis was performed between these isolates.

### Ethics statements

This study did not include human participants, and thus the need for review and approval from the institutional ethics committee was waived, in accordance with “Ethical Guidelines for Medical and Health Research Involving Human Subjects” [29, 30]. If epidemiological information is linked to bacterial strains isolated from human stool, the research would fall under the remit of guidelines on studies featuring human participants. The strains analyzed here were isolated from human stool samples collected by public health centers in Chiba and Yamagata prefectures in accordance with the Act on Prevention of Infectious Diseases and Medical Care for Patients Suffering Infectious Diseases (Act No. 114 of 1998). When we used the strains in this study, no associated epidemiological information, other than whether the strains were derived from sporadic cases, outbreak cases, or intra-family cases, was available; that is, the strains had already been anonymized at the personal data by public health centers.

In this study, ownership of the strains was transferred in writing to the study authors by the individuals infected with O157 via the public health centers. Generally, ownership of a bacterial strain in human stool is thought to belong to the test facility where the strain was isolated; therefore, this study did not officially require this transfer procedure. Nevertheless, in this study, written informed consent was obtained from the infected individuals to protect their rights. For minors, consent was obtained from their parents or guardians.

## Results

### Subdivision of O157 strains into clades

A total of 136 Chiba isolates were subdivided into five major clades by SNP and LSPA-6 analyses as follows: 21 strains into clade 2; 26 strains into clade 3; 29 strains into clade 7; 30 strains into clade 8; and 30 strains into clade 12 (S2 Table). All strains were subdivided into the major clades. In this study, all clades other than the major clades were defined as minor clades (i.e., clade 1, descendant clade 4/5; ancestral clades 4/5, 6, and 9; and putative clades 10, 11, and 13).

### Analyses of minimum spanning tree and linkage disequilibrium of TR patterns obtained by MLVA

**Fig 1:**
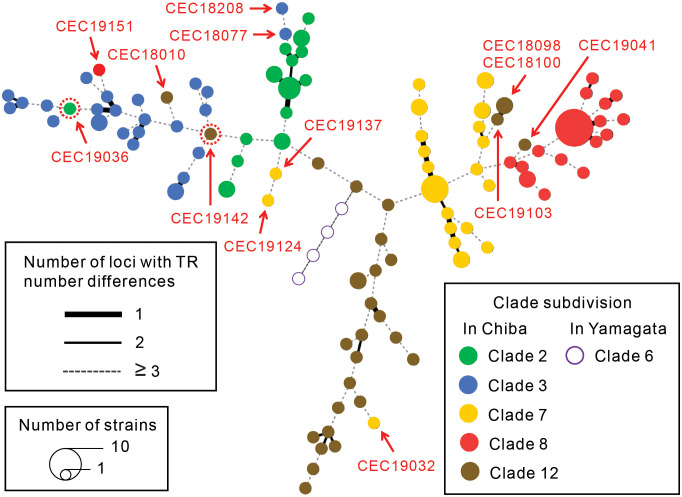
Minimum spanning tree (MST) reconstructed from multi-locus variable-number tandem repeat analysis data. MST was reconstructed using all enterohemorrhagic *Escherichia coli* O157 (O157) strains isolated in Chiba prefecture. Then, all clade 6 strains isolated in Yamagata prefecture were plotted in the MST. Colors of nodes in the MST indicate clade subdivision of these strains by both single-nucleotide polymorphism analysis and LSPA-6. Red arrows indicate O157 strains in which homoplasy was observed on tandem repeat (TR) patterns. The strains surrounded by red dotted lines were subdivided incorrectly into clade 3 when using a maximum *a posteriori* estimation formula.

An MST analysis of the MLVA data revealed that most Chiba isolates were clustered on branches separate from the clades determined by the IS-printing and LSPA-6 data, indicating that the strains in each clade based on the MLVA data had unique TR patterns (Fig 1). In addition, homoplasy of TR patterns was observed for some strains in each clade (Fig 1; S2 Table), specifically, one strain in clade 2, two strains in clade 3, three strains in clade 7, one strain in clade 8, and six strains in clade 12 were not clustered with other strains in the same clade. However, the *I*^*S*^_*A*_ values in each clade of the Chiba isolates were significantly different from zero (Table 1), indicating linkage disequilibrium in the TR patterns of each clade.

**Table 1:** Linkage disequilibrium for tandem repeats in each major clade using all strains isolated in Chiba prefecture.

Clade	*V*_*D*_	*V*_*e*_	*I* ^*S*^_*A*_	Monte Carlo simulation		
				*Var* (*V*_D_)	*p*	*L*
**2**	10.8020	2.5248	0.2049	0.2185	<0.001	3.3666
**3**	4.3491	2.7113	0.0377	0.1401	<0.001	3.3783
**7**	8.9899	2.5804	0.1552	0.1323	<0.001	3.1786
**8**	7.7885	2.3885	0.1413	0.1739	<0.001	3.1387
**12**	4.6914	2.3856	0.0604	0.0445	<0.001	2.7329

### Construction of a MAP estimation formula for putative subdivision of O157 strains into clades using MLVA data

The distribution rates of TR numbers in the MLVA results were investigated for each locus in each major clade using the Chiba isolates to construct a likelihood database for MAP estimation (Table 2; S3 Table; S1 Fig). There was no or very low diversity in five of the 17 loci (i.e., EH111-11, EH111-14, EH111-8, EH26-7, and EHC-6) among the major clade strains; that is, almost all or all strains had no gene amplifications by MLVA in these loci. This is consistent with previous reports [3, 31], indicating that the lack of amplification in this study would not have been caused by the failure of multiplex PCR, but was rather due to the absence of locus. The remaining 12 loci showed a certain amount of diversity, but there were many TRs with zero distribution; for example, no strains showed a TR of 1 in EHC 1. The existence of TRs with zero distribution indicated the need to address the zero-frequency problem. Therefore, all the distribution ratios in each clade were subjected to weighted smoothing, and a likelihood database was constructed (S4 Table).

**Table 2:** Quartile values of the number of tandem repeats at each locus in each major clade using all strains isolated in Chiba prefecture ^a^.

Locus	Clade 2	Clade 3	Clade 7	Clade 8	Clade 12
**EH26-7**	0, 0, 0	0, 0, 0	0, 0, 0	0, 0, 0	0, 0, 0
**EH111-11**	2, 2, 2	2, 2, 2	2, 2, 2	2, 2, 2	2, 2, 2
**EH111-14**	0, 0, 0	0, 0, 0	0, 0, 0	0, 0, 0	0, 0, 0
**EH111-8**	1, 1, 1	1, 1, 1	1, 1, 1	1, 1, 1	1, 1, 1
**EH157-12**	4, 4, 4	4, 4, 4	4, 4, 4	6, 6, 6	1, 1, 2
**EHC-1**	5, 5, 5	6, 6, 6	7.25, 11, 11	9.25, 12, 12	6, 7, 7
**EHC-2**	4, 4, 4	4, 4, 4	5, 5, 5	4, 4, 4	5, 5, 6.75
**EHC-5**	0, 0, 0	0, 2, 10	0, 0, 0	0, 0, 0	0, 0, 7
**EHC-6**	0, 0, 0	0, 0, 0	0, 0, 0	0, 0, 0	0, 0, 0
**O157-3**	9, 9, 12	9, 10, 10	4, 4, 8	11.5, 15, 15	0, 7, 7.75
**O157-9**	12, 12, 16	7.75, 9.5, 12	0, 0, 10	9, 9, 10	9, 10, 11
**O157-17**	7, 7, 8	7, 7, 8	5, 5, 6.75	4, 4, 4	3, 3, 3
**O157-19**	6, 6, 6	5, 6, 6	7, 7, 7	7, 7, 7	5, 6, 6
**O157-25**	5, 7, 8	4, 4, 5	3, 3, 3	5, 5, 5	3, 4, 5
**O157-34**	12, 12, 12	12, 12, 12	9, 9, 9	9, 9, 9	8.25, 9, 9
**O157-36**	3, 3, 5	6, 6, 6	4, 6, 6	9, 9, 9	5, 6, 7
**O157-37**	6, 6, 7	6.75, 7, 8	5, 6, 7	5, 5, 6	6, 7, 8

^a^ Three numbers (e.g., “5, 7, 8” in clade 2 at O157-25) indicate the 25^th^, 50^th^, and 75^th^ percentile values when the numbers of tandem repeats at each locus in each major clade in the strains are arranged in ascending order.

### CR of clade subdivision via MAP estimation versus SNP and lineage analyses

For the Chiba isolates, the CRs for clades 3, 7, and 8 were 100%, and those for clades 2 and 12 were >95% (Table 3(A); S2 Table). Of the 13 strains with homoplasy, 11 were subdivided into the correct clades, and the remaining two strains (one strain each in clades 2 and 12) were incorrectly subdivided into clade 3 (Fig 1; S2 Table). These two strains were also clustered with clade 3 strains on an MST (Fig 1).

**Table 3:** Putative subdivision of major clade strains into clades by maximum *a posteriori* estimation from multilocus variable-number tandem repeat analysis (MLVA) data.

**(A) Chiba prefecture**							
**Major clade**	**Putative clade based on MLVA data**					**Total**	**Concordance ratio (%)**
	**2**	**3**	**7**	**8**	**12**		
**2**	20	1	0	0	0	21	95.2
**3**	0	26	0	0	0	26	100.0
**7**	0	0	29	0	0	29	100.0
**8**	0	0	0	30	0	30	100.0
**12**	0	1	0	0	29	30	96.7
**(B) Yamagata prefecture**							
**2**	15	4	0	0	0	19	78.9
**3**	2	31	0	0	0	33	93.9
**7**	0	0	8	0	1	9	88.9
**8**	0	0	0	8	1	9	88.9
**12**	0	0	0	0	5	5	100.0

For the major clades of the Yamagata isolates, the CRs for clade 12 were 100%, and those for clades 3, 7, and 8 were approximately 90% (Table 3(B); S2 Table). Four strains in clade 2 were incorrectly subdivided into clade 3, and the CR for clade 2 was the lowest among all CRs of the Chiba and Yamagata isolates (Table 3(A) and 3(B); S2 Table). No significant differences were observed among the CRs between Chiba and Yamagata isolates of the same clade. For the minor clade Yamagata isolates, one strain in clade 1 was incorrectly subdivided into clade 2, one strain in descendant clade 4/5 was placed in clade 3, and four clade 6 strains were placed in clade 12 (Table 4; S2 Table).

**Table 4:** Putative subdivision of minor clade strains into clades by maximum *a posteriori* estimation from multilocus variable-number tandem repeat analysis (MLVA) data.

Minor clade	Putative clade based on MLVA data					Total
	2	3	7	8	12	
**1**	1	0	0	0	0	1
**Des 4/5** ^a^	0	1	0	0	0	1
**6**	0	0	0	0	4	4

^a^ Descendant clade 4/5.

## Discussion

This study established a method for the putative subdivision of O157 strains into clades by MAP estimation using MLVA data. Owing to its superiority as a method for epidemiological analysis, MLVA has been universally adopted by public institutes laboratories for the surveillance of O157 strain infections in Japan. However, the analysis has been used previously only to determine whether O157 strains were derived from the same clone. The present study increases the value of MLVA as a tool, showing that it is also useful as a method with low laboratory burden for subdividing O157 strains into phylogenetic groups, which could lead to the enhancement O157 strain surveillance in Japan.

Supporting evidence of the validity of our findings includes the very high CRs (88.9%−100%) for all major clades of the Chiba and Yamagata isolates, with the exception of clade 2 Yamagata isolates. In this study, a likelihood database for each major clade was constructed using the Chiba isolates; from this database, an MAP estimation formula was constructed. It is expected that the CRs for a strain set used to construct a database would be higher than those for a strain set that is not used for database construction. Out of the 13 Chiba isolates with homoplasy, 11 strains were correctly subdivided into clades by MAP estimation, which indicates that if a strain with homoplasy is included in the database, it is likely to be assigned to the correct clade by the MAP estimation. This phenomenon may have contributed to the higher CRs found in this study. However, the CRs obtained here for Chiba and Yamagata isolates of the same clade were not significantly different, indicating that the dataset for Chiba isolates used in this study had little influence on the evaluation of the accuracy of the MAP estimation.

Subdivision of some strains with homoplasy in MST into correct clades using MAP estimation would be due to the difference in algorithms between the MST and MAP estimation. Briefly, MST is constructed by connecting strains with the same number of TRs when comparing TRs in each of 17 loci. In other words, when two strains have more loci with the same number of TRs, these strains are closer in the MST. In MAP estimation, the posterior probabilities for each clade in a strain are calculated by continuously multiplying likelihoods of TRs in each of 17 loci. Then, the strain is determined to belong to the clade giving the largest probability. As an example, the genetic distance from strain B to strain A is compared with that from strain C to strain A using MST analysis and MAP estimation. Two loci in MLVA differ between strains A and B, and likelihoods of TRs in strain B for strain A in the two loci are very low. Meanwhile, three loci in MLVA differ between strains A and C, and likelihoods of TRs in strain C for strain A in the three loci are high. In this case, the genetic distance in MST between strains A and B is shorter than that between strains A and C. However, in MAP estimation, the posterior probability that strain C belongs to strain A is higher than the probability that strain B belongs to strain A; that is, strain C is genetically closer to strain A than to strain B in the MAP estimation. Thus, the two different algorithms in MST and MAP estimation evaluate genetic distances differently even using the same strains, which would further cause the phenomenon that strains with homoplasy in the MST were subdivided into correct clades using MAP estimation. This phenomenon indicates the added value of the MAP estimation.

The MAP estimation method of this study will contribute to research elucidating O157 clade distributions in various regions of Japan, if clade subdivision using MLVA data is widely adopted by public institutes. Previously, we demonstrated similar clade distributions in three widely separated areas of Japan [18], but distributions in other areas of Japan have not been investigated yet. A few previous studies confirmed that there are different distributions of phylogenetic groups of O157 strains between different areas of a single country, e.g., the North and South Islands of New Zealand [32, 33]. As part of the Japanese surveillance, most local public institutes perform MLVA for the early detection of O157 strain outbreaks [1]. If the MLVA data obtained by these institutes can be utilized for the MAP estimation method, the O157 clade distribution of Japan will be revealed in entirety. If the O157 strain distributions differ by region, the risk of O157 strain infection may also differ. Investigations of the O157 clade distributions are considered to be important for public health.

Further global comparisons of O157 clade distributions may become possible if the MAP estimation method becomes widely used by overseas institutes. Although O157 clade distributions have been reported to differ among countries [5, 18, 34], these distributions were revealed in only a few countries. The MLVA for O157 strains is used in many countries, especially European countries and the USA [35, 36]. Therefore, this MAP estimation method may play an important role in elucidating O157 clade distributions worldwide. However, slight modifications to this MAP estimation method may be needed for overseas institutes because the numbers of MLVA loci used by other regions differ from those used by Japan and the CDC’s PulseNet (S5 Table) [2, 3, 20].

A limitation of this study is that it could not sufficiently demonstrate whether minor clade O157 strains (clade 1, descendant or ancestral clade 4/5, clade 6, and clade 9) can be correctly subdivided into clades by MAP estimation. However, our previous study [18] reported that the minor clades, other than clade 6, accounted for very few O157 strains (0% to 2%) in Japan. Therefore, this limitation may not seriously affect O157 clade distribution surveillance using MAP estimation in Japan. As for clade 6, all such strains were incorrectly subdivided into clade 12 by the MAP estimation. The relatively small number of clade 6 strains [18] may also have influenced this result. Clade 6 strains were reported to be more pathogenic than the strains of other clades [8]; therefore, further studies are necessary to demonstrate whether minor clade strains can be correctly subdivided by MAP estimation. This study investigated the TRs and clades of all analyzed O157 strains, in addition to the likelihood database of TRs (S2 and S4 Tables), and this limitation could be resolved if those researching O157 strains update the likelihood table in this study with their MLVA data.

## Conclusion

This study successfully established a method to subdivide O157 strains into clades by MAP estimation from MLVA data. The dataset constructed using the Chiba isolates may greatly assist with O157 clade subdivision.

## Supporting information

S1 Fig Distribution of tandem repeats (TRs) at each locus in a multi-locus variable-number tandem repeat analysis for major clades of enterohemorrhagic *E*. *coli* O157 strains. The decimal point was truncated when a 25^th^–75^th^ percentile value had a decimal point. (PPTX)

S1 Table Differences in clade classifications of enterohemorrhagic *E*. *coli* O157 by Manning et al. [5] and Hirai et al. [4]. (XLSX)

S2 Table Details of enterohemorrhagic *E*. *coli* O157 strains analyzed in this study, and putative subdivision of these strains into clades by maximum *a posteriori* (MAP) estimation from multilocus variable-number tandem repeat analysis (MLVA) data. (XLSX)

S3 Table Distribution rates of the numbers of tandem repeats (TRs) in each locus of a multilocus variable-number tandem repeat analysis (MLVA) for each major clade of enterohemorrhagic *E*. *coli* O157 strains isolated in Chiba prefecture. (XLSX)

S4 Table Likelihood database of tandem repeats (TRs) in each locus of a multilocus variable-number tandem repeat analysis (MLVA) for major clades using enterohemorrhagic *E*. *coli* O157 strains isolated in Chiba prefecture. (XLSX)

S5 Table Differences in the analyzed loci and used primers between the multilocus variable-number tandem repeat analysis (MLVA) performed by Japanese public institutes and the method recommended by CDC’s PulseNet. (XLSX)
