# Supplementary material for: Another advantage of multi-locus variable-number tandem repeat analysis that can putatively subdivide enterohemorrhagic Escherichia coli O157 strains into clades by maximum a posteriori estimation
Source: PLoS One. 2023 Mar 30;18(3):e0283684. doi: 10.1371/journal.pone.0283684 (PMC10062581; doi:10.1371/journal.pone.0283684)
Supplement: S1 Fig — (PPTX) [file pone.0283684.s001.pptx]

## Slide 1
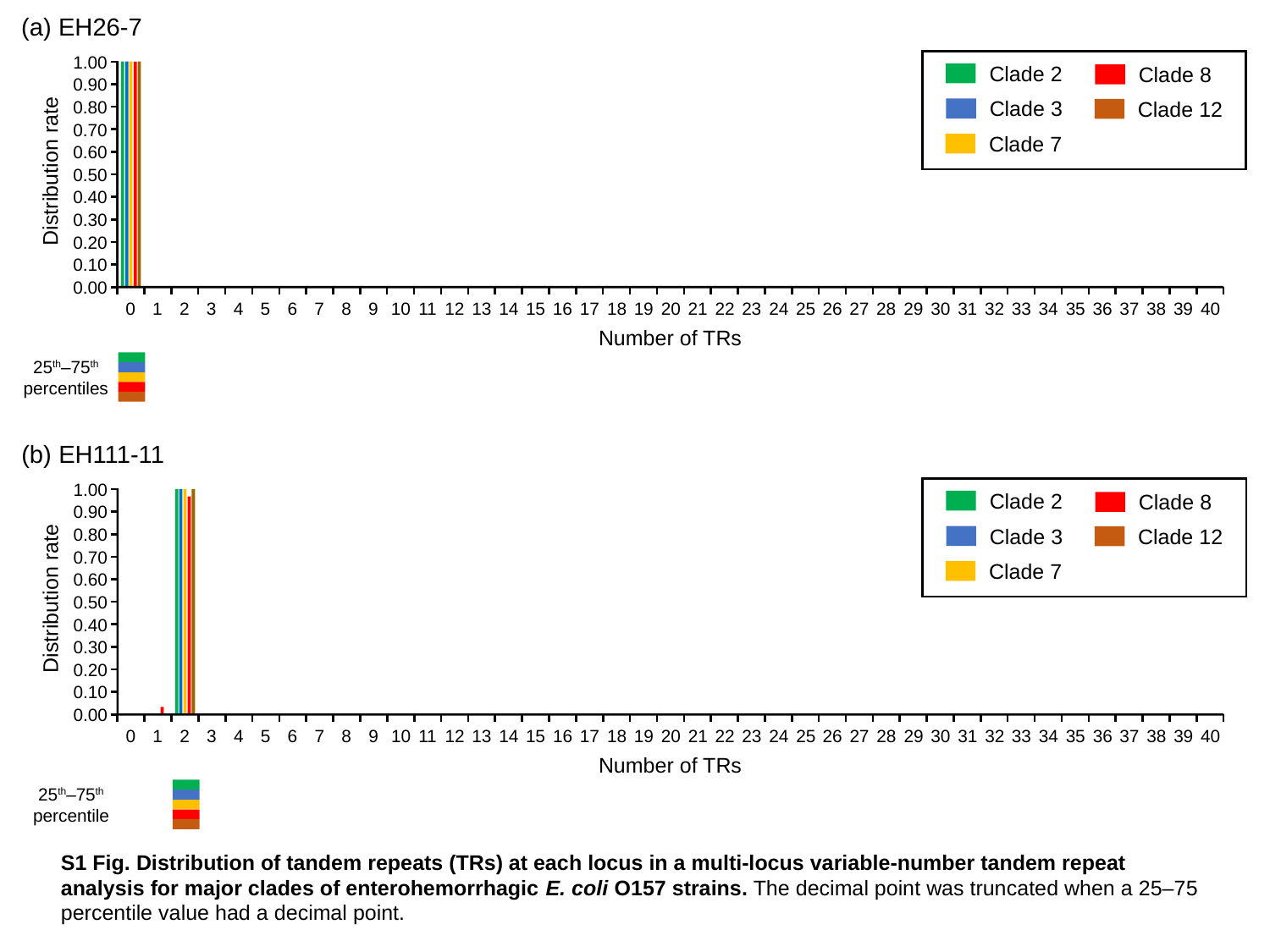

(a) EH26-7
1.00
Clade 2
Clade 8
0.90
Clade 3
Clade 12
0.80
0.70
Clade 7
0.60
Distribution rate
0.50
0.40
0.30
0.20
0.10
0.00
0
1
2
3
4
5
6
7
8
9
10
11
12
13
14
15
16
17
18
19
20
21
22
23
24
25
26
27
28
29
30
31
32
33
34
35
36
37
38
39
40
Number of TRs
25th–75th percentiles
(b) EH111-11
1.00
Clade 2
Clade 8
0.90
Clade 3
Clade 12
0.80
0.70
Clade 7
0.60
Distribution rate
0.50
0.40
0.30
0.20
0.10
0.00
0
1
2
3
4
5
6
7
8
9
10
11
12
13
14
15
16
17
18
19
20
21
22
23
24
25
26
27
28
29
30
31
32
33
34
35
36
37
38
39
40
Number of TRs
25th–75th percentile
S1 Fig. Distribution of tandem repeats (TRs) at each locus in a multi-locus variable-number tandem repeat analysis for major clades of enterohemorrhagic E. coli O157 strains. The decimal point was truncated when a 25–75 percentile value had a decimal point.

## Slide 2
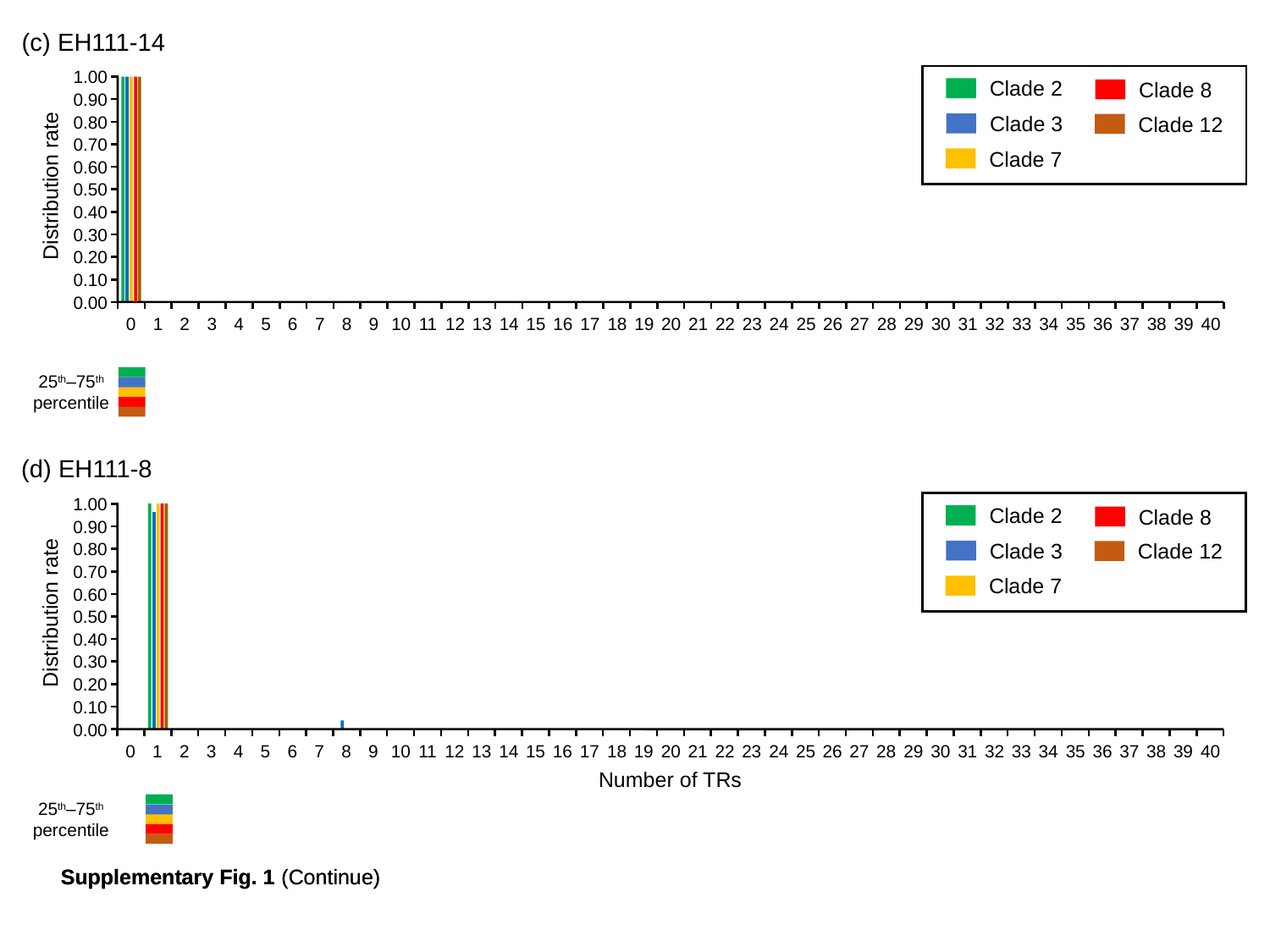

(c) EH111-14
1.00
Clade 2
Clade 8
0.90
Clade 3
Clade 12
0.80
0.70
Clade 7
0.60
Distribution rate
0.50
0.40
0.30
0.20
0.10
0.00
0
1
2
3
4
5
6
7
8
9
10
11
12
13
14
15
16
17
18
19
20
21
22
23
24
25
26
27
28
29
30
31
32
33
34
35
36
37
38
39
40
25th–75th percentile
(d) EH111-8
1.00
Clade 2
Clade 8
0.90
Clade 3
Clade 12
0.80
0.70
Clade 7
0.60
Distribution rate
0.50
0.40
0.30
0.20
0.10
0.00
0
1
2
3
4
5
6
7
8
9
10
11
12
13
14
15
16
17
18
19
20
21
22
23
24
25
26
27
28
29
30
31
32
33
34
35
36
37
38
39
40
Number of TRs
25th–75th percentile
Supplementary Fig. 1 (Continue)
Supplementary Fig. 1 (Continue)

## Slide 3
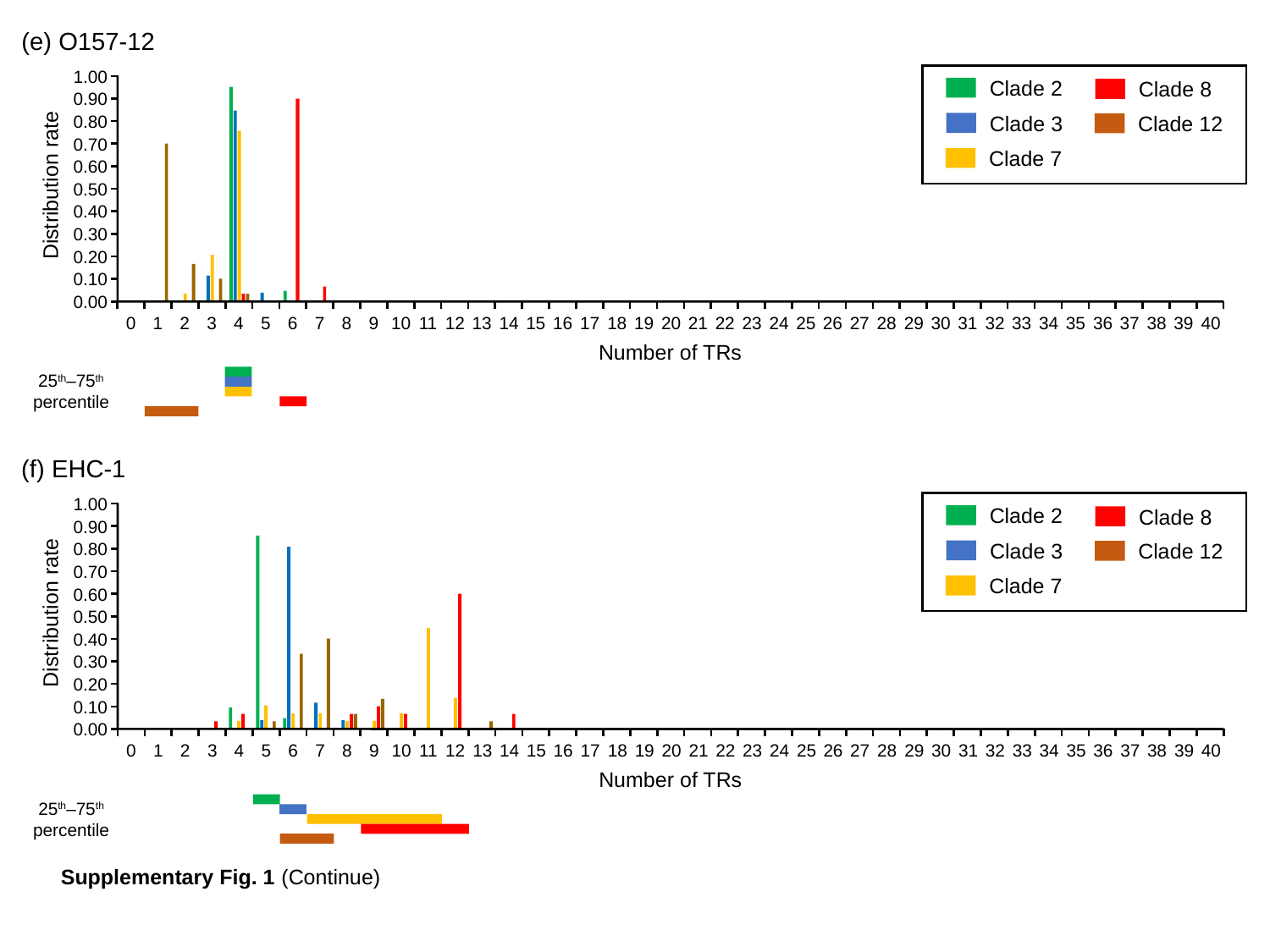

(e) O157-12
1.00
Clade 2
Clade 8
0.90
Clade 3
Clade 12
0.80
0.70
Clade 7
0.60
Distribution rate
0.50
0.40
0.30
0.20
0.10
0.00
0
1
2
3
4
5
6
7
8
9
10
11
12
13
14
15
16
17
18
19
20
21
22
23
24
25
26
27
28
29
30
31
32
33
34
35
36
37
38
39
40
Number of TRs
25th–75th percentile
(f) EHC-1
1.00
Clade 2
Clade 8
0.90
Clade 3
Clade 12
0.80
0.70
Clade 7
0.60
Distribution rate
0.50
0.40
0.30
0.20
0.10
0.00
0
1
2
3
4
5
6
7
8
9
10
11
12
13
14
15
16
17
18
19
20
21
22
23
24
25
26
27
28
29
30
31
32
33
34
35
36
37
38
39
40
Number of TRs
25th–75th percentile
Supplementary Fig. 1 (Continue)

## Slide 4
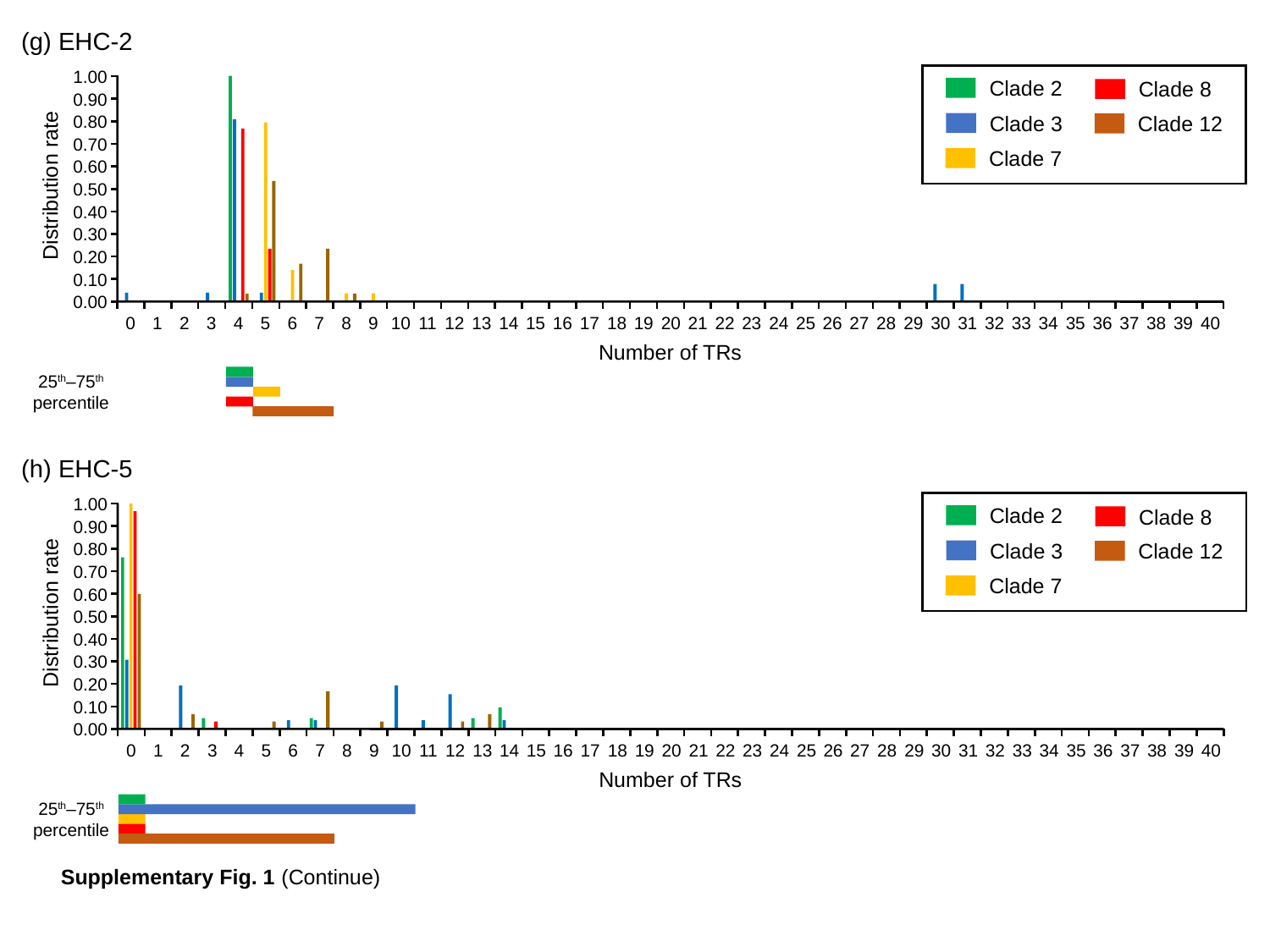

(g) EHC-2
1.00
Clade 2
Clade 8
0.90
Clade 3
Clade 12
0.80
0.70
Clade 7
0.60
Distribution rate
0.50
0.40
0.30
0.20
0.10
0.00
0
1
2
3
4
5
6
7
8
9
10
11
12
13
14
15
16
17
18
19
20
21
22
23
24
25
26
27
28
29
30
31
32
33
34
35
36
37
38
39
40
Number of TRs
25th–75th percentile
(h) EHC-5
1.00
Clade 2
Clade 8
0.90
Clade 3
Clade 12
0.80
0.70
Clade 7
0.60
Distribution rate
0.50
0.40
0.30
0.20
0.10
0.00
0
1
2
3
4
5
6
7
8
9
10
11
12
13
14
15
16
17
18
19
20
21
22
23
24
25
26
27
28
29
30
31
32
33
34
35
36
37
38
39
40
Number of TRs
25th–75th percentile
Supplementary Fig. 1 (Continue)

## Slide 5
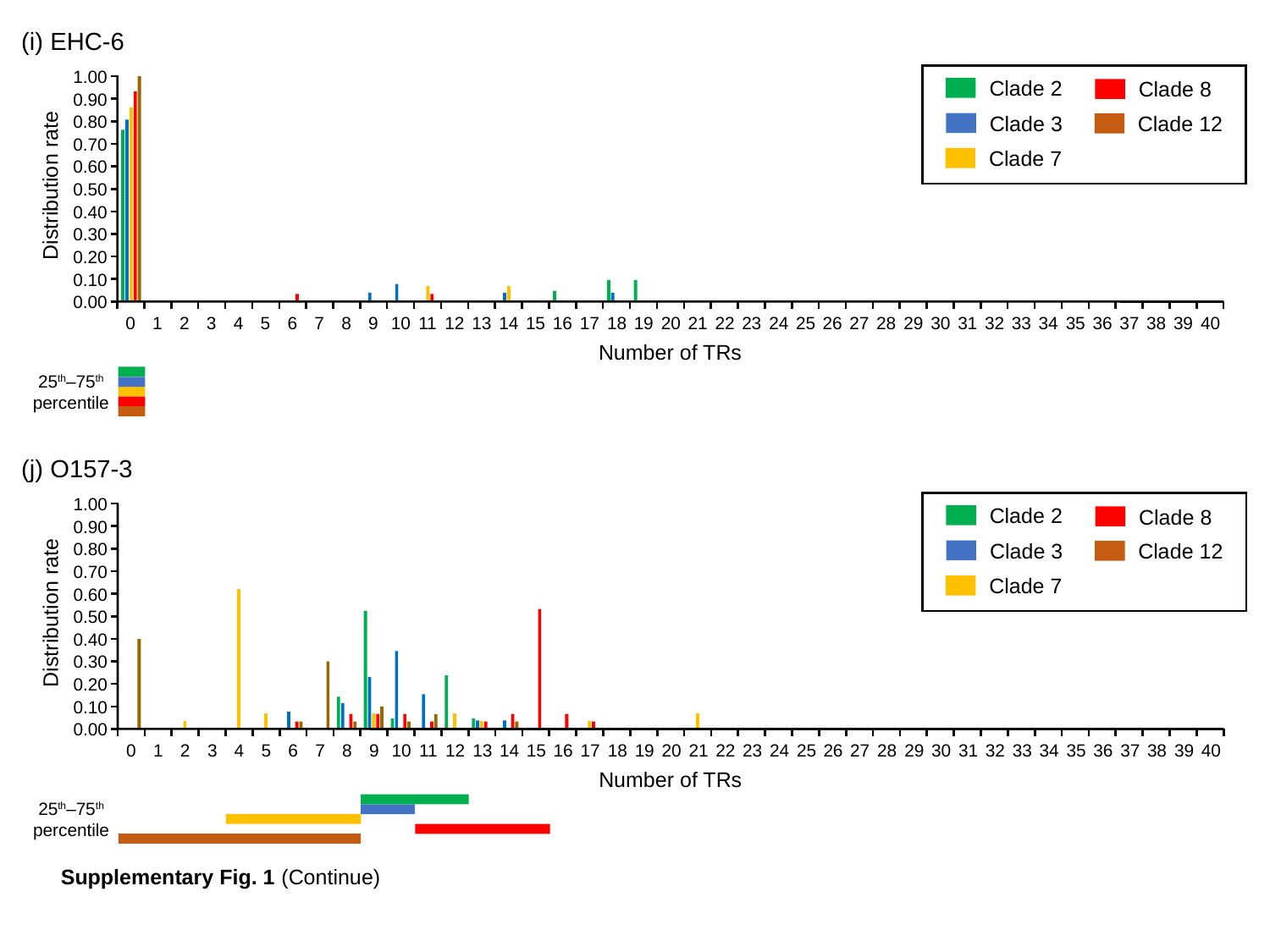

(i) EHC-6
1.00
Clade 2
Clade 8
0.90
Clade 3
Clade 12
0.80
0.70
Clade 7
0.60
Distribution rate
0.50
0.40
0.30
0.20
0.10
0.00
0
1
2
3
4
5
6
7
8
9
10
11
12
13
14
15
16
17
18
19
20
21
22
23
24
25
26
27
28
29
30
31
32
33
34
35
36
37
38
39
40
Number of TRs
25th–75th percentile
(j) O157-3
1.00
Clade 2
Clade 8
0.90
Clade 3
Clade 12
0.80
0.70
Clade 7
0.60
Distribution rate
0.50
0.40
0.30
0.20
0.10
0.00
0
1
2
3
4
5
6
7
8
9
10
11
12
13
14
15
16
17
18
19
20
21
22
23
24
25
26
27
28
29
30
31
32
33
34
35
36
37
38
39
40
Number of TRs
25th–75th percentile
Supplementary Fig. 1 (Continue)

## Slide 6
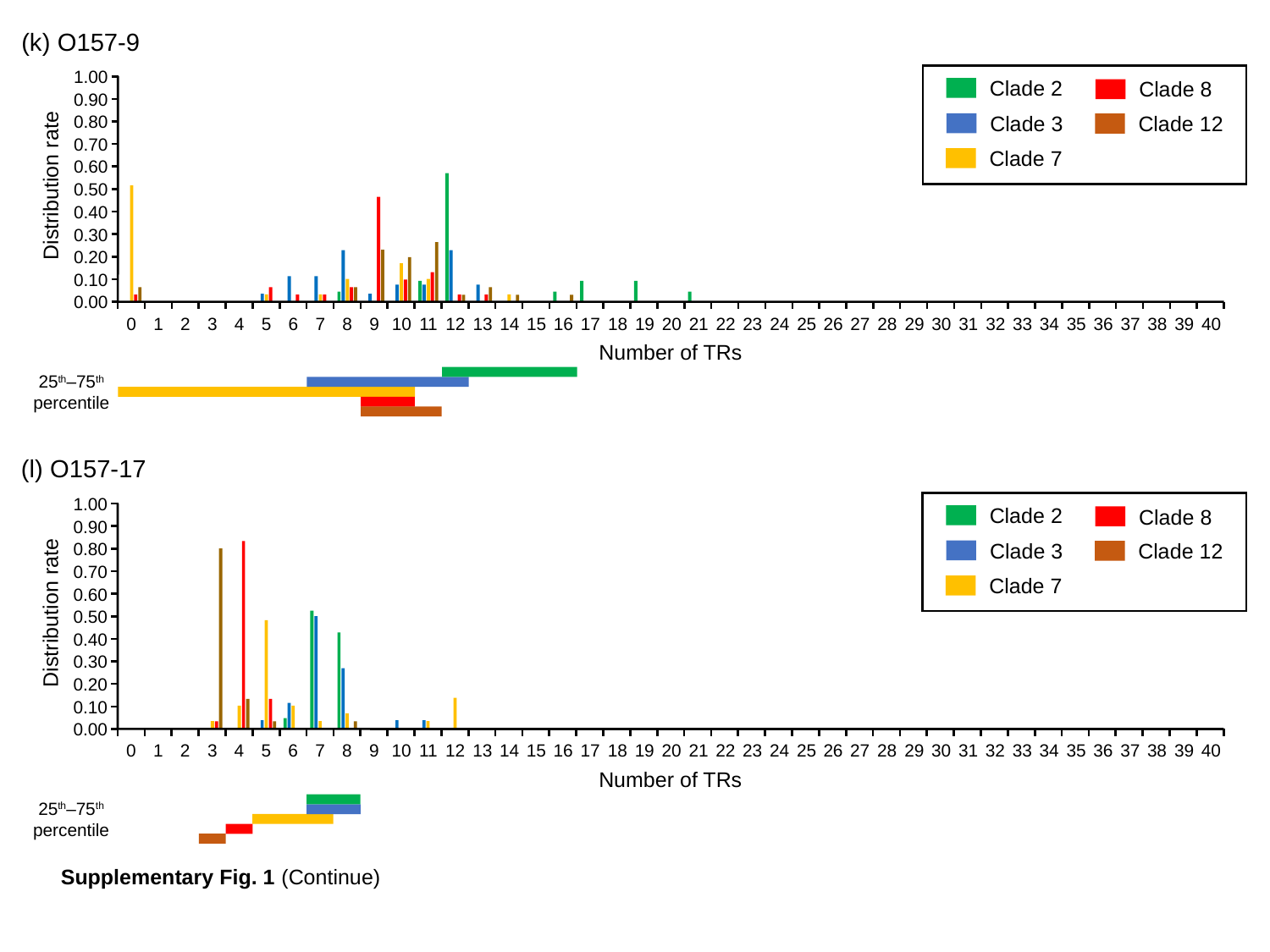

(k) O157-9
1.00
Clade 2
Clade 8
0.90
Clade 3
Clade 12
0.80
0.70
Clade 7
0.60
Distribution rate
0.50
0.40
0.30
0.20
0.10
0.00
0
1
2
3
4
5
6
7
8
9
10
11
12
13
14
15
16
17
18
19
20
21
22
23
24
25
26
27
28
29
30
31
32
33
34
35
36
37
38
39
40
Number of TRs
25th–75th percentile
(l) O157-17
1.00
Clade 2
Clade 8
0.90
Clade 3
Clade 12
0.80
0.70
Clade 7
0.60
Distribution rate
0.50
0.40
0.30
0.20
0.10
0.00
0
1
2
3
4
5
6
7
8
9
10
11
12
13
14
15
16
17
18
19
20
21
22
23
24
25
26
27
28
29
30
31
32
33
34
35
36
37
38
39
40
Number of TRs
25th–75th percentile
Supplementary Fig. 1 (Continue)

## Slide 7
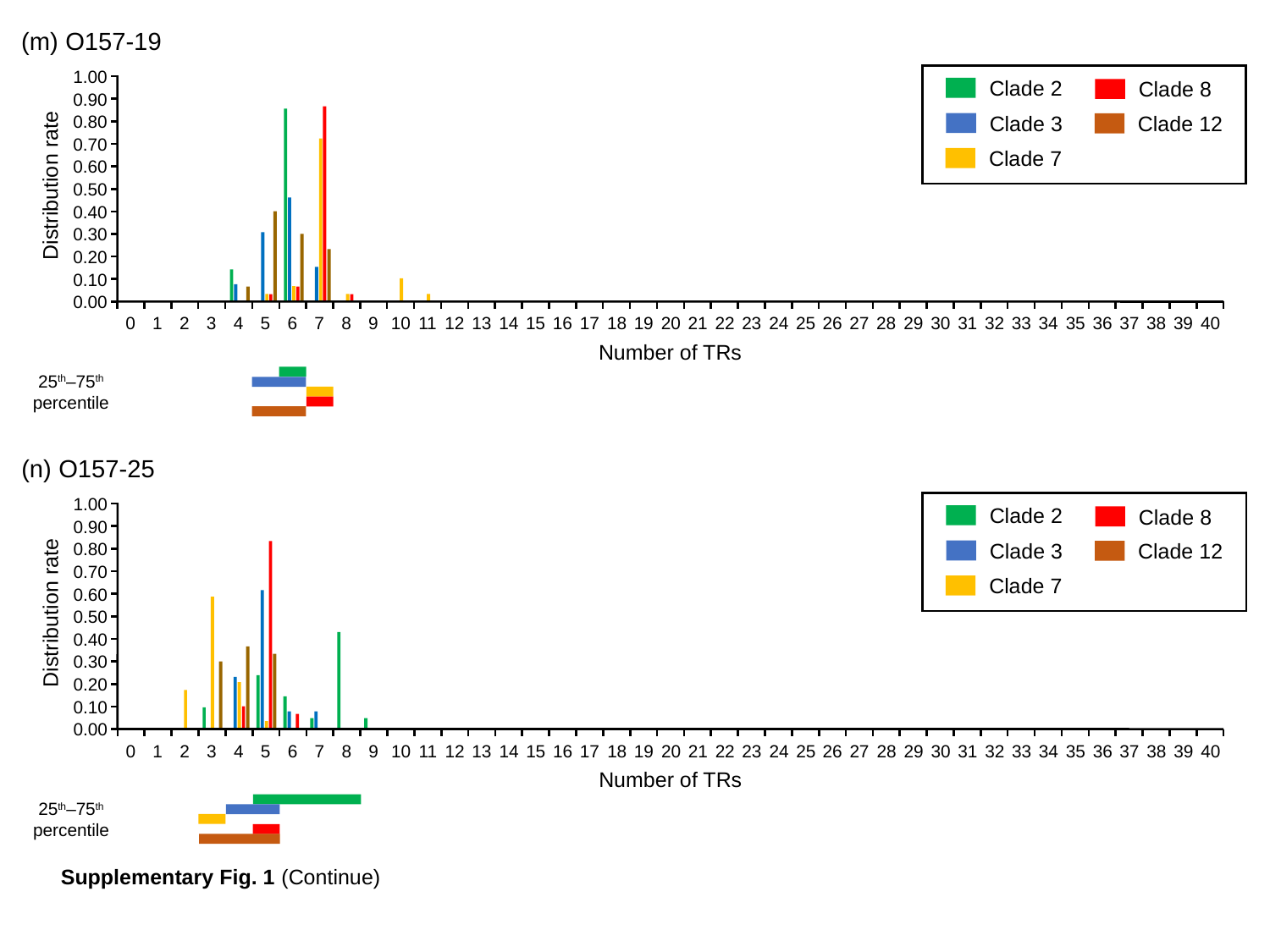

(m) O157-19
1.00
Clade 2
Clade 8
0.90
Clade 3
Clade 12
0.80
0.70
Clade 7
0.60
Distribution rate
0.50
0.40
0.30
0.20
0.10
0.00
0
1
2
3
4
5
6
7
8
9
10
11
12
13
14
15
16
17
18
19
20
21
22
23
24
25
26
27
28
29
30
31
32
33
34
35
36
37
38
39
40
Number of TRs
25th–75th percentile
(n) O157-25
1.00
Clade 2
Clade 8
0.90
Clade 3
Clade 12
0.80
0.70
Clade 7
0.60
Distribution rate
0.50
0.40
0.30
0.20
0.10
0.00
0
1
2
3
4
5
6
7
8
9
10
11
12
13
14
15
16
17
18
19
20
21
22
23
24
25
26
27
28
29
30
31
32
33
34
35
36
37
38
39
40
Number of TRs
25th–75th percentile
Supplementary Fig. 1 (Continue)

## Slide 8
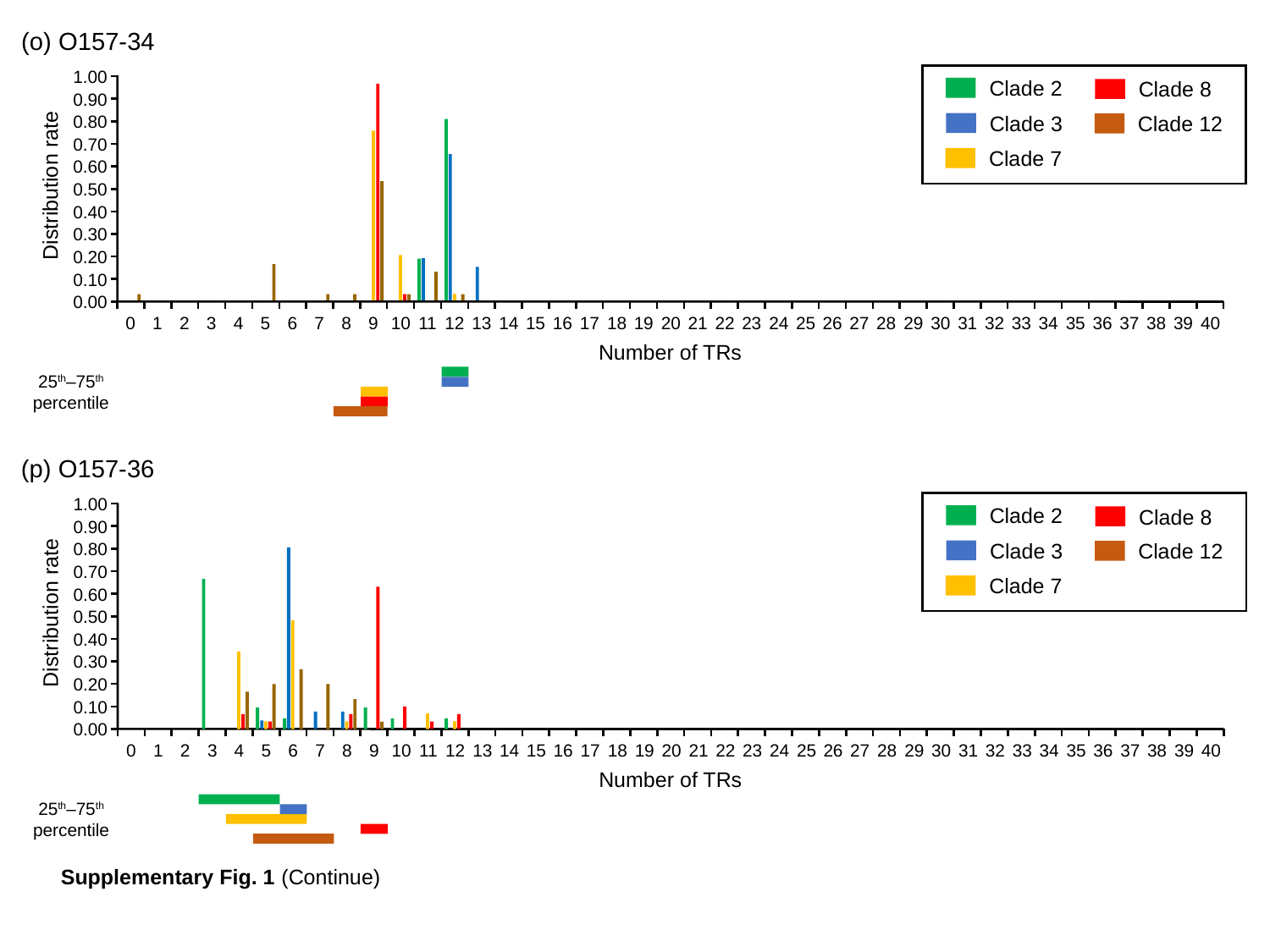

(o) O157-34
1.00
Clade 2
Clade 8
0.90
Clade 3
Clade 12
0.80
0.70
Clade 7
0.60
Distribution rate
0.50
0.40
0.30
0.20
0.10
0.00
0
1
2
3
4
5
6
7
8
9
10
11
12
13
14
15
16
17
18
19
20
21
22
23
24
25
26
27
28
29
30
31
32
33
34
35
36
37
38
39
40
Number of TRs
25th–75th percentile
(p) O157-36
1.00
Clade 2
Clade 8
0.90
Clade 3
Clade 12
0.80
0.70
Clade 7
0.60
Distribution rate
0.50
0.40
0.30
0.20
0.10
0.00
0
1
2
3
4
5
6
7
8
9
10
11
12
13
14
15
16
17
18
19
20
21
22
23
24
25
26
27
28
29
30
31
32
33
34
35
36
37
38
39
40
Number of TRs
25th–75th percentile
Supplementary Fig. 1 (Continue)

## Slide 9
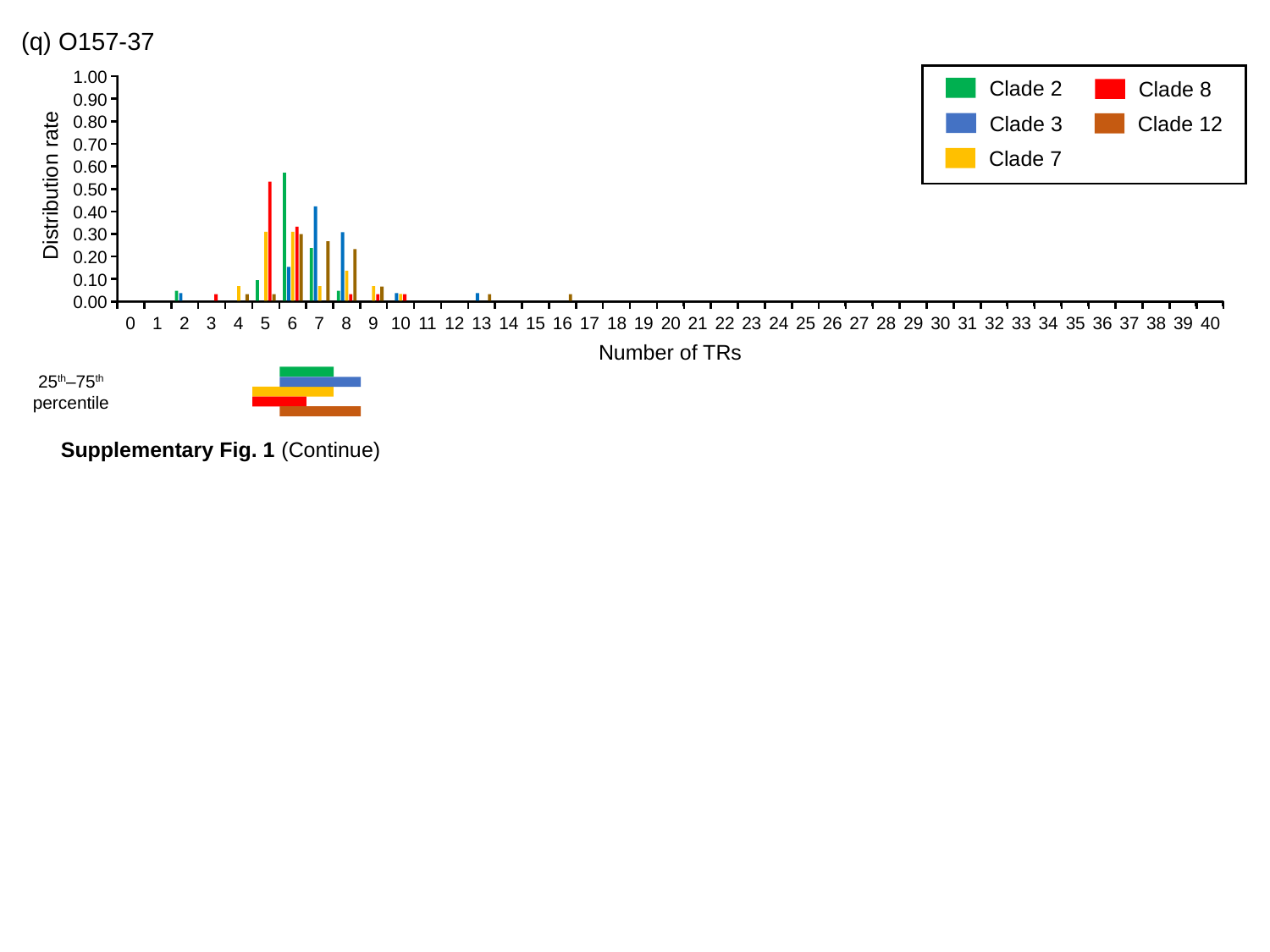

(q) O157-37
1.00
Clade 2
Clade 8
0.90
Clade 3
Clade 12
0.80
0.70
Clade 7
0.60
Distribution rate
0.50
0.40
0.30
0.20
0.10
0.00
0
1
2
3
4
5
6
7
8
9
10
11
12
13
14
15
16
17
18
19
20
21
22
23
24
25
26
27
28
29
30
31
32
33
34
35
36
37
38
39
40
Number of TRs
25th–75th percentile
Supplementary Fig. 1 (Continue)
